# Viral Infection Correlates with a Better Clinical Outcome than Pulmonary Exacerbation of Bacterial Origin in Paediatric Patients with Cystic Fibrosis

**DOI:** 10.3390/pathogens14090850

**Published:** 2025-08-26

**Authors:** Zuzanna Stachowiak, Marta Andrzejewska, Katarzyna Jończyk-Potoczna, Beata Narożna, Anna Musiał, Anna Wiesner, Anna Bręborowicz, Aleksandra Szczepankiewicz, Irena Wojsyk-Banaszak

**Affiliations:** 1 Molecular and Cell Biology Unit, Poznań University of Medical Sciences, 60-572 Poznań, Poland; bnarozna@ump.edu.pl (B.N.); alszczep@ump.edu.pl (A.S.); 2 Department of Paediatric Pulmonology, Allergy and Clinical Immunology, Poznań University of Medical Sciences, 60-572 Poznań, Poland; awiesner@ump.edu.pl (A.W.); ambreborowicz@ump.edu.pl (A.B.); iwojsyk@ump.edu.pl (I.W.-B.); 3 Department of Pediatric Oncology, Hematology and Transplantation, Poznań University of Medical Sciences, 60-572 Poznań, Poland; marta.andrzejewska.rm@gmail.com; 4 Doctoral School, Poznan University of Medical Sciences, 60-812 Poznań, Poland; 5 Department of Pediatric Radiology, Poznań University of Medical Sciences, 60-572 Poznań, Poland; jonczyk@ump.edu.pl; 6 Department of Pediatric Diabetes, Auxology and Obesity, Poznań University of Medical Sciences, 60-572 Poznań, Poland; anna.musial@ump.edu.pl; 7 Centre of Experimental Medicine, Poznań University of Medical Sciences, 60-806 Poznań, Poland

**Keywords:** cystic fibrosis, respiratory viral infection, pulmonary exacerbation, Shwachman–Kulczycki score, Brasfield score

## Abstract

Cystic fibrosis is a progressive disease affecting various organs of the human body, including the respiratory system. While the effect of bacterial infection on cystic fibrosis outcome has been comprehensively described, little is known about the impact of viruses. We collected 49 nasopharyngeal swabs derived from cystic fibrosis paediatric patients during pulmonary exacerbation and tested them for the presence of respiratory viruses to elucidate the influence of the viral infection on their clinical outcome. We found that patients infected with a virus, compared to those in whom molecular testing for viruses was negative, are characterised by a better clinical outcome, as measured by the Shwachman–Kulczycki score (*p* = 0.006) and have better chest radiographs, as indicated by the Brasfield score (*p* = 0.002). Moreover, these patients have lower C-reactive protein levels (*p* = 0.002). We assume this unexpected association of better clinical outcomes during viral infection should be studied further.

## 1. Introduction

Cystic fibrosis (CF) is an autosomal recessive, progressive, multi-organ disease primarily affecting the lungs and gastrointestinal functions. It is caused by pathogenic variants of the cystic fibrosis transmembrane regulator (*CFTR*) gene. In the course of the disease, a gradual deterioration of pulmonary function is observed, triggered by recurrent pulmonary exacerbations primarily due to multiple infections [1,2,3,4,5,6].

The lung parameters’ deterioration is also provoked by a chronic infection with pathogens such as *Staphylococcus aureus*, especially methicillin-resistant strains and/or *Pseudomonas aeruginosa* [1,7]. Moreover, the CF airways are often colonised by other microorganisms, including anaerobic bacteria and fungi [8,9]. A bacterial infection is frequently preceded by a viral infection that sensitises the pulmonary epithelium and allows colonisation [10,11]. While Gram-negative bacteria are believed to be a major factor in pulmonary function deterioration, multiple viral infections are also regarded as contributing, but the data are still scarce [12,13,14,15,16].

As the disease is characterised by a continuous worsening of patients’ pulmonary status, the clinical outcome of cystic fibrosis should be monitored [17,18]. This may be done using a wide range of scales and scores. The Shwachman–Kulczycki (SK) score is one of the parameters assessed by a physician that proves effective in measuring CF severity, while the Brasfield score is calculated based on findings in the chest radiograph of CF patients [19,20].

The prevalence of viral infection differs in the literature. It accounts for from 13% to 52% of pulmonary exacerbations, according to van Ewijk et al., or from 5% to 68%, according to Hizal et al. [21,22]. Viral infections may also be clinically silent and detected during routine control visits as an accidental finding [12,23].

Currently, little is known about the impact of viral infection on the clinical outcome of paediatric patients diagnosed with cystic fibrosis. There are only a few papers that describe this topic; however, most of these studies were performed in small sample sizes [24,25,26]. Moreover, there is a lack of studies on Central European CF patients looking for the correlation between viral infection and clinical outcomes. The primary aim of our study was to assess the impact of viral respiratory infection on pulmonary exacerbation and how it affects the clinical state of paediatric patients with CF.

## 2. Materials and Methods

### 2.1. Study Design

We performed a cross-sectional observational study and retrospectively analysed the microbiological status of children with cystic fibrosis based on 49 nasopharyngeal swabs obtained from girls and boys aged 0.5–17 years. All patients were admitted to the Department of Pulmonology, Paediatric Allergy and Immunology during pulmonary exacerbation (PE) in the time period between February 2014 and December 2021. The diagnosis of PE was made according to the EuroCareCF working group definition, as an episode in a patient with CF that prompted a prescription for a new antibiotic therapy by a treating physician [27]. We excluded patients with SARS-CoV-2 infection.

In the study group, we collected the data from the current PE episode and from a previous scheduled visit for clinical follow-up, during which patients had no clinical symptoms or laboratory abnormalities characteristic of exacerbation (stable condition). The study was approved by the Local Bioethics Committee at Poznan University of Medical Sciences (no. 386/17).

### 2.2. Clinical Analysis

All patients were previously diagnosed with CF based on clinical presentation, two positive sweat chloride tests, and the biallelic presence of pathogenic variants of the *CFTR* gene, and the diagnosis was reviewed according to the current guidelines [28]. The inclusion criterion was a performed nasopharyngeal swab for molecular analyses of the genetic material of 21 respiratory viruses and bacteria causing pulmonary infections, as well as complete blood count, C-reactive protein, sputum culture results, and lung function tests (spirometry, body plethysmography, and impulse oscillometry). Moreover, a chest radiograph (Brasfield score) was assessed, and the disease severity was evaluated with the Shwachman–Kulczycki score.

### 2.3. PCR Testing for Respiratory Viruses and Bacteria Detection

All nasopharyngeal swabs were sent for molecular analyses using Multiplex PCR and the FTD Respiratory Pathogens 25 panel (Fast Track Diagnostics, Junglinster, Luxembourg). The presence of the following viruses and bacteria was analysed: influenza A; AH1N1 and B; rhinovirus; coronaviruses NL63, 29E, OC43, and HKU; parainfluenza 1, 2, 3, and 4; metapneumovirus A and B; bocavirus; respiratory syncytial virus A and B; adenovirus; enterovirus; parechovirus; *Mycoplasma pneumoniae*; *Chlamydia pneumoniae*; *Streptococcus pneumoniae*; *Haemophilus influenzae*; and *Staphylococcus aureus*.

### 2.4. Statistical Analysis

Statistical univariate analyses were performed with the Statistica package v.13 (v.12, Statsoft, Krakow, Poland) and PQStat (PQStat v.1.8.2.208, PQStat, Poznań, Poland ). Normal distribution was checked using the Shapiro–Wilk test. For normally distributed data, parametric tests were used (Student’s *t*-test), while data without normal distribution were analysed using nonparametric tests (Mann–Whitney U test or Chi-squared test). The comparison of the examined parameters in two groups of patients (Shwachman–Kulczycki score, Brasfield score, and lung parameters) was performed using Student’s *t*-test or the Mann–Whitney U test with correction for continuity for independent variables. *p*-value < 0.05 was considered statistically significant.

## 3. Results

### 3.1. Clinical Description of the Studied Group

For research purposes, we collected 49 nasopharyngeal swabs from 26 individuals with CF during PEs to investigate the presence of respiratory viruses. Nine patients experienced more than one exacerbation during the study period. The age of the studied patients ranged from 0.5 to 17 years, with a median age of 10 years. Overall, 20 children were homozygous for the F508del (40.8%) variant, 18 were heterozygous for the F508del (36.7%) variant, and 11 had other pathogenic variants (22.4%). For the whole PE group, the median Shwachman–Kulczycki score was 65, and the median Brasfield score was 13. All patients with a detected virus were positive for bacteria in the microbiological analysis of sputum, mainly *Staphylococcus aureus*. Thus, both groups with PE (with viral infection and without viral infection) were similar in terms of microbial status (Supplementary Table S1).

Further clinical description of the studied group, including CF-associated complications, is available in Table 1. Material was also collected from CF patients in stable conditions; the characteristics are shown in Table 2.

### 3.2. Results of Epidemiological Analysis

**Figure 1:**
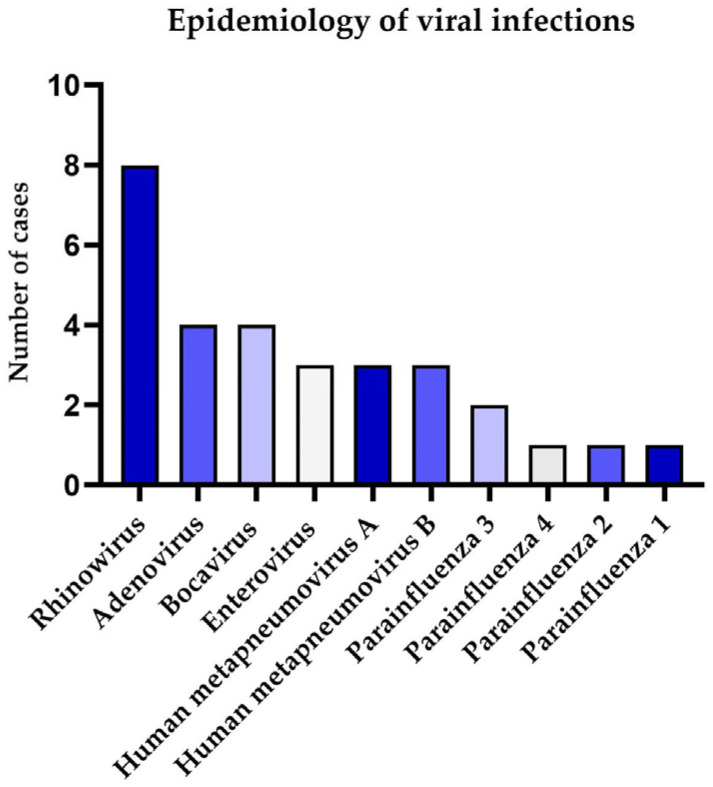
Epidemiology of viral infection among paediatric cystic fibrosis patients in pulmonary exacerbation.

Out of 49 tested nasopharyngeal swabs, 19 (39%) were positive for the presence of at least one respiratory virus. Six samples (12%) were positive for the presence of more than one virus, and one sample was positive for the presence of four viruses. Overall, the swabs were positive in total for 10 viruses, with the most common virus being rhinovirus, *n* = 8 (42%), followed by adenovirus *n* = 4 (26%), bocavirus *n* = 4 (21%), enterovirus *n* = 3 (16%), human metapneumovirus A *n* = 3 (16%), human metapneumovirus B *n* = 3 (16%), parainfluenza 3 *n* = 2 (10,5%), parainfluenza 4 *n* = 1 (5%), parainfluenza 1 *n* = 1 (5%), and parainfluenza 2 *n* = 1 (5%). Figure 1 depicts the number of samples positive for the respective viruses.

**Figure 2:**
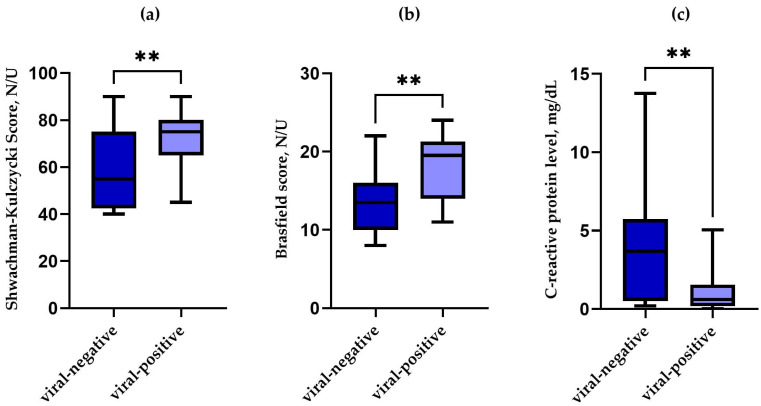
Box and whisker plot showing the relationship between the presence of the virus and: the Shwachman–Kulczycki score (**a**); the Brasfield score (**b**); the C-reactive protein level (**c**) among CF paediatric patients with PE. N/U—no units, **—*p* ≤ 0.01.

When we compared the presence of respiratory viral infection in CF patients during exacerbation, we found a correlation between the presence of any virus and a significantly higher Shwachman–Kulczycki score (*p* = 0.006) (Figure 2a), a higher Brasfield score (*p* = 0.002) (Figure 2b), and lower CRP (*p* = 0.002) (Figure 2a) as compared to the patients during exacerbation without viral infection. Patients positive for viruses also tended to be younger.

We did not find any statistically significant differences in the lung function parameters and microbiological results in the patients positive vs. negative for viral infection during PE.

**Figure 3:**
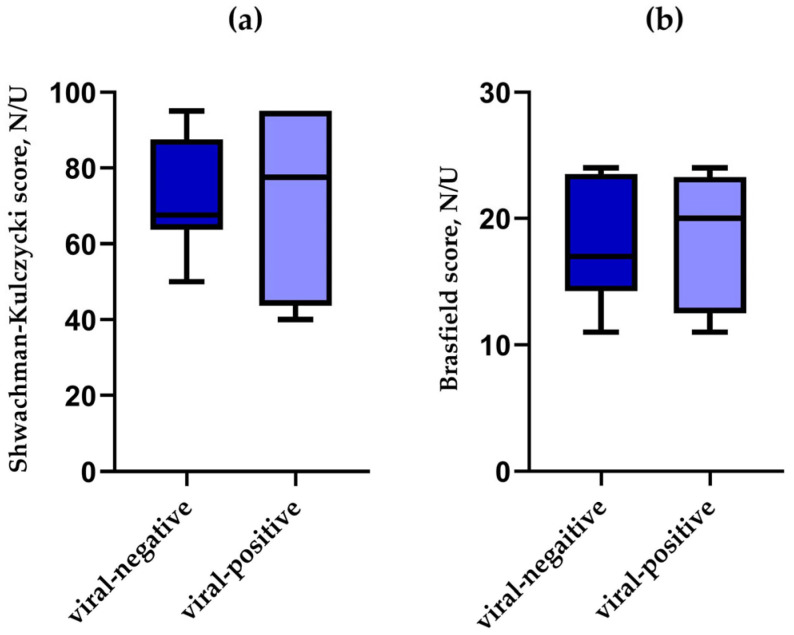
Box and whisker plot showing Shwachman–Kulczycki scores (**a**) and Brasfield score (**b**) among CF paediatric patients in a stable state that preceded PE, in which they were assessed for virus infection. N/U—no units.

We additionally checked if the patients with viral infection were in a generally better state than those in whom molecular tests for the presence of respiratory viruses were negative, which might have influenced the results. To do so, we evaluated the associated medical history of the above-described individuals (Table 2). Unfortunately, data from a preceding visit in the outpatient clinic without any PE were not available in all cases of the 49 visits with PE. We found 16 such patients in a stable state, and we evaluated their status before PE. N = 6 (37.5%) had viral infection at the time, and all patients were colonised with bacteria, predominantly *S. aureus*. The patients in stable condition with a viral infection still had an overall better (albeit not significantly) median Shwachman–Kulczycki score of 77.5, with a minimum of 55 and a maximum of 85 (no virus group—60, min. 40, max. 80), *p* = 0.106 (Figure 3a), but a similar median Brasfield score, i.e., 17 (min. 11, max. 24), as compared to the no-virus group with a median of 20 (min. 11, max. 24), *p* = 0.999 (Figure 3b).

## 4. Discussion

The most important message of our study is that the isolation of a respiratory virus in paediatric CF patients during PE is, surprisingly, associated with less severe clinical outcomes than PE in patients with no molecular evidence of viral infection, as indicated by Shwachman–Kulczycki and Brasfield scores, as well as CRP levels in these patients. Such results were obtained as early as 1989 by Ramsey et al., who described lower severity scores in CF patients with viral infection and concluded they were unable to prove the adverse effect of viral infection on the pulmonary function of the patients [29]. Similarly, another study showed that respiratory viruses do not impact the severity of CF but contribute to further infection acquisition [30]. Later on, the studies revolved around bacteria’s impact. Only recently did the data grow vastly with the COVID-19 pandemic triggering the interest in the accelerated use of PCR testing for viruses [31]. Hizal et al. described a similarly unexpected relation recently, with no deterioration in pulmonary function in CF patients who underwent viral infection [22]. We noticed better SK and Brasfield scores, which correlate with better clinical outcomes in patients with viral infection vs. those not infected by respiratory viruses during PE. The direct reason for such dependence has not been elucidated yet.

Meyer et al. showed that virus-positive and virus-negative groups of children with CF do not differ in terms of disease severity or bacterial colonisation [32]. However, a study performed among Indian paediatric patients indicated that the viral infection precedes a more severe bacterial infection and possibly contributes to adverse outcomes in the patients. These patients had worse Shwachman–Kulczycki scores, required more intensive antimicrobial therapy, including intravenous administration, and had higher mortality over time [33]. This shows possible risks to our patients, despite earlier, better outcomes in PE. Our research showed that virus-positive stable patients had better SK and Brasfield scores than virus-negative individuals, though the difference was not statistically significant. These surprising results may be explained by more frequent medical contact prompted by viral infection symptoms and implementation of more aggressive symptomatic treatment, as well as antibacterial therapies (due to coexisting bacterial colonisation), leading to improvement of clinical condition. It is essential to stress that in CF patients, the assessment of a single-microbial agent in the pathogenesis of PE or as a contributing factor to better outcomes is challenging, given the multiple colonisations in the whole respiratory tract. It has been recently described that it is not apparent if Streptococcal infection in people with CF impacts the severity of bronchopulmonary disease, especially when factors such as the co-existence of other pathogens, deregulation of host immunity, or epithelial condition have to be taken into account [34]. We speculate that such deep connections may also be relevant to the results we describe with the current bacterial co-infection, but it is not possible to support this with relevant literature, as research on this topic has not yet advanced.

Epidemiological data concerning the prevalence of viral infection are conflicting regarding the status of people with CF, their baseline treatment, and the geographical origin. In a study by Scherz et al., approximately half of the patients had a viral infection [35]. The group described the epidemiology, with the most common being rhinovirus, with a prevalence of 26.5%. Hamed et al. performed a cross-sectional observational study on 60 CF paediatric patients, with PE, detecting a virus in 80% of them, with the most common being rhinovirus again (43.4%), which is consistent with our results, as 42% of our patients had rhinovirus infection, and other research in this field [32,36,37]. Other frequent viruses in our patients include adenovirus (26%) and bocavirus (21%), similar to Hamed’s work, with a 13.3% and 20% prevalence, respectively. We also found a similar rate of multiple viral infections of 12% as other groups (10.4–16.7%) [35,36].

Conversely, we had a much lower percentage of viruses present during exacerbation than Hamed and colleagues—39%—but our number is similar to the results obtained by Gonzalez-Rosales et al., who detected a virus in 34% of CF paediatric patients undergoing PE [38]. This study also indicated that such patients do better, having higher FEV_1_ and shorter hospitalisation time [38]. Scherz and colleagues made similar conclusions in relation to rhinovirus infection and FEV_1_ trajectories [35]_._ In our cohort, however, FEV_1_ was not significantly related to the presence of viral infection.

Pulmonary exacerbations in the course of CF display different phenotypic features characterised by biomarker profile, clinical presentation, and outcomes. Most of our patients presented with low inflammatory indices that would categorise them into a pauci-inflammatory subphenotype according to Carter et al. [39]. This low inflammatory response, especially evident in the group with viral infections, might be partly responsible for the better outcome of the aforementioned group of patients. Unfortunately, due to the retrospective design of our study, we did not evaluate more specifically inflammatory markers other than C-reactive protein, white blood cell, and neutrophil count.

There is a growing body of knowledge on the molecular mechanisms of how viruses act on the airways of individuals with CF [12,40]. Interestingly, this association is multifaceted as it not only allows for subsequent bacterial colonisation/infection and further pulmonary exacerbations but also may contribute to the deterioration of pulmonary function over time, as indicated by spirometry and body plethysmography. Interesting conclusions were drawn by the group of Kartsiouni et al., who systematically reviewed non-CF bronchiectasis and did not confirm that the presence of viruses was related to pulmonary symptoms [41]. We believe viral–pulmonary and viral–bacterial interactions may also occur in CF patients. The role of viruses disturbing the airway microenvironment and augmenting the inflammatory process may lead to further deterioration in patients’ clinical state. The research on the molecular level has shown that the viral dependence on the activity of CFTR channels is impaired in CF. Chloride channels are predominantly targeted in viral infection as they resemble viroporins. Influenza infection leads to reduced activity of airway CFTR by slowing its maturation, leading to a decrease in its function [42,43,44]. Another interesting explanation of viral impact on CFTR function is the inhibition of its cyclic nucleotidase and cAMP reduction, hence the accession of pathogens and higher prevalence of further viral infections [42,45]. In light of these data, we postulate that molecular mechanisms of other viruses–airway crosstalk should be sought to elucidate the association of better outcomes described by us and Gonzalez-Rosales et al. [38]. Regarding rhinovirus, which is the most abundant in many studied groups, its effect has been studied on the nasal mucosa of CF patients, showing higher expression of the CFTR protein, but also its inhibition and altered function. In parallel, epithelial sodium channel function rises. Both channels’ function has adverse effects, such as increased mucus viscosity and susceptibility to subsequent bacterial infections. The group concludes that more studies need to be done to explain it [46]. The results they yielded seem to oppose the better outcomes of virus-positive CF patients.

The limitation of our study is the fact that this is a single-centre study based on retrospective findings and a relatively small cohort of patients. The molecular studies for the presence of viruses were performed in an external reference laboratory. Though we manage many PE CF patients, only a small percentage of them are tested for viruses with the use of this comprehensive panel, leading to a possible bias in the results, as we could incorporate only the patients who were tested in our study. Importantly, this study does not take into consideration the effect of *CFTR* modulators (CFTRm) as they were not implemented in our country during the studied period. Despite the increasing availability of CFTR modulators in many countries, there are still many people with CF who are not eligible due to the reimbursement policies or their genetic background [47].

The evidence of the CFTRm impact on the incidence of viral infections in children remains an area of investigation [48]. In vitro studies did not show the restoration of antiviral immune mechanisms in bronchial epithelial cells from people with CF treated with ivacaftor and lumacaftor [49]. The STOP2 study, investigating PE in adults with CF, showed a higher incidence of PE caused by viruses in adults with CF treated with CFTRm than in those who did not receive CFTRm. The authors postulated that this shift towards viral infections might be caused by a decrease in bacterial infections, which is well documented in people with CF on CFTRm [17,50]. In a multicentre study, Fireizen and colleagues found a decrease in the incidence of viral infections from 2018 to 2022 (when highly effective CFTRm were introduced) in children and young adults who were hospitalised because of PE. Many of the hospitalised children did not receive CFTRm, so these data reflect a global trend rather than individual responses of patients treated with modulators [51]. The changing epidemiology of viral infections in the era of modulator treatment, especially in the paediatric population, needs further research and longitudinal studies.

## 5. Conclusions

To conclude, we found an unexpected association between better clinical outcomes and radiology findings as indicated by the concentrations of C-reactive protein, Shwachman–Kulczycki, and Brasfield scores in paediatric CF patients with pulmonary exacerbation and a diagnosed viral infection, as compared to CF patients with PE and no respiratory viruses identified. We assume this is rather an interesting dependence, not a causal link, that should be studied further to elucidate the impact of viral infection on cystic fibrosis patients.

## Figures and Tables

**Table 1:** Characteristics of the studied group of CF patients during pulmonary exacerbation.

Variable	PE	Viral-Negative	Viral-Positive	*p*-Value
Number of samples	49	30	19	-
Sex, female	22 (44.9%)	12 (40.0%)	10 (52.6%)	-
Median age, years (range)	10 (0.5–17)	11 (2–17)	6 (0.5–17)	0.002
F508del homozygous	20 (40.8%)	10 (33.3%)	10 (52.6%)	1.000
F508del heterozygous	18 (36.7%)	9 (30.0%)	9 (47.4%)	1.000
Other variants	11 (22.4%)	11 (36.7%)	0	0.003
Median body mass index, BMI, kg/m^2^ (range)	14.81 (11.2–21.53)	14.93 (13.33–20.9)	14.79 (11.2–21.53)	0.700
Pancreatic insufficiency	43 (87.8%)	27 (90.0%)	16 (84.0%)	0.547
Median SK score (range)	65 (40–90)	57.5 (40–90)	75 (45–90)	0.006
Median Brasfield score (range)	15 (8–24)	13.5 (8–22)	19 (11–24)	0.002
Median oxygen saturation, %	92.0 (50.1–99.5)	90.7 (56.6–99.0)	94.3 (50.1–99.5)	0.209
Median C-reactive protein level, mg/dL, (range)	1.50 (0.02–13.75)	3.68 (0.20–13.75)	0.61 (0.02–5.05)	0.002
Median absolute neutrophil count, 10^3^/mm^3^ (range)	6.68 (1.33–24.28)	6.70 (1.33–17.39)	5.08 (1.58–24.28)	0.312
Mean forced expiratory volume in the first second, FEV_1_ z-score (SD)	−2.02 (±1.82)	−1.99 (±1.82)	−2.12 (±1.88)	0.840
Median Resonant Frequency, Fres (range)	19.74 (11.45–31.37)	19.71 (11.45–31.37)	21.54 (12.37–29.47)	0.639
Median reactance at 10 Hz % due value, 10x due value (range)	212 (−2748–3493)	240 (−2748–3493)	185.5 (−66–427)	0.577
Median area of reactance, AX (range)	1.60 (0.22–5.14)	1.43 (0.22–5.14)	1.79 (0.32–4.45)	0.566
Mean total lung capacity, TLC (SD)	4.29 (±1.14)	4.52 (±1.10)	3.94 (±1.17)	0.077

Abbreviation: SD—standard deviation.

**Table 2:** Characteristics of the studied group of CF patients during the stable stage of disease.

Variable	Stable	Viral-Negative	Viral-Positive	*p*-Value
Number of samples	16	10	6	-
Sex, female	10 (62.5%)	8 (80.0%)	2 (33.3%)	-
Mean age, years (SD)	8.43 (±4.20)	8.65 (±4.40)	8.08 (±4.21)	0.804
F508del homozygous	7 (43.8%)	4 (40.0%)	3 (50.0%)	0.696
F508del heterozygous	6 (37.5%)	4 (40.0%)	2 (33.3%)	0.790
Other variants	3 (18.7%)	2 (20.0%)	1 (16.7%)	0.869
Mean body mass index, BMI, kg/m^2^ (SD)	15.16 (±2.27)	15.41 (±2.46)	14.17 (±2.06)	0.591
Pancreatic insufficiency	15 (93.8%)	9 (90.0%)	6 (100%)	0.790
Median SK score (range)	70 (40–85)	60 (40–80)	77.5 (55–85)	0.106
Median Brasfield score (range)	18 (11–24)	17 (11–24)	20 (11–24)	0.999
Median oxygen saturation, %	90.65 (47.7–96.6)	92.1 (47.7–96.6)	87.9 (73–96.6)	0.476
Median C-reactive protein level, mg/dL, (range)	0.2 (0.02–4.57)	0.2 (0.02–1.57)	0.71 (0.03–4.57)	0.196
Mean absolute neutrophil count, 10^3^/mm^3^ (SD)	4.50 (±2.45)	4.70 (±2.23)	4.19 (±2.95)	0.950
Median forced expiratory volume in the first second, FEV_1_ z-score (range)	−2.46 (−4.46–1.2)	−2.26 (−4.46–(−0.10))	−2.58 (−2.58–1.2)	0.897
Mean Resonant Frequency, Fres (SD)	22.73 (±6.97)	22.40 (±5.69)	23.34 (±9.61)	0.819
Median reactance at 10 Hz % due value, 10x due value (range)	212 (−365–1577)	192 (−365–1577)	212 (−47–270)	1.000
Mean area of reactance, AX (SD)	2.262 (1.554)	2.306 (1.703)	2.184 (1.425)	0.895
Median total lung capacity, TLC (range)	4.78 (2.96–6.03)	5.22 (2.96–6.03)	4.53 (4.38–4.68)	0.487

Abbreviation: SD—standard deviation.

## Data Availability

The datasets presented in this article are not readily available because the data include sensitive medical data of paediatric patients. Requests to access the anonymised datasets should be directed to the Department of Pulmonology, Pediatric Allergy and Clinical Immunology, Szpitalna St. 27/33, 60-572 Poznan.

## Supplementary Materials

The following supporting information can be downloaded at: https://www.mdpi.com/article/10.3390/pathogens14090850/s1, Table S1: Pulmonary pathogen characteristics of patients included in the study.

## Abbreviations

The following abbreviations are used in this manuscript:

CF	Cystic fibrosis
CFTR	Cystic fibrosis transmembrane regulator
SK	Shwachman–Kulczycki (score)
PE	Pulmonary exacerbation
BMI	Body mass index
SD	Standard deviation
FEV_1_	Forced expiratory volume in the first second
FRES	Resonant Frequency
AX	Area of reactance
TLC	Total lung capacity

## References

[1] Sanders D.B., Bittner R.C.L., Rosenfeld M., Hoffman L.R., Redding G.J., Goss C.H. (2010). Failure to recover to baseline pulmonary function after cystic fibrosis pulmonary exacerbation. Am. J. Respir. Crit. Care Med..

[2] Sanders D.B., Hoffman L.R., Emerson J., Gibson R.L., Rosenfeld M., Redding G.J., Goss C.H. (2010). Return of FEV1 after pulmonary exacerbation in children with cystic fibrosis. Pediatr. Pulmonol..

[3] Goss C.H. (2019). Acute Pulmonary Exacerbations in Cystic Fibrosis. Crit. Care Med..

[4] Emerson J., Rosenfeld M., McNamara S., Ramsey B., Gibson R.L. (2002). *Pseudomonas aeruginosa* and other predictors of mortality and morbidity in young children with cystic fibrosis. Pediatr. Pulmonol..

[5] Konstan M.W., Morgan W.J., Butler S.M., Pasta D.J., Craib M.L., Silva S.J., Stokes D.C., Wohl M.E.B., Wagener J.S., Regelmann W.E. (2007). Risk Factors For Rate of Decline in Forced Expiratory Volume in One Second in Children and Adolescents with Cystic Fibrosis. J. Pediatr..

[6] Waters V., Ratjen F. (2015). Pulmonary exacerbations in children with cystic fibrosis. Ann. Am. Thorac. Soc..

[7] Aaron S.D., Ramotar K., Ferris W., Vandemheen K., Saginur R., Tullis E., Haase D., Kottachchi D., Denis M.S., Chan F. (2004). Adult cystic fibrosis exacerbations and new strains of *Pseudomonas aeruginosa*. Am. J. Respir. Crit. Care Med..

[8] Blanchard A.C., Waters V.J. (2019). Microbiology of Cystic Fibrosis Airway Disease. Semin. Respir. Crit. Care Med..

[9] Caverly L.J., Vandevanter D.R. (2022). The Elusive Role of Airway Infection in Cystic Fibrosis Exacerbation. J. Pediatr. Infect. Dis. Soc..

[10] Van Ewijk B.E., Wolfs T.F., Aerts P.C., Van Kessel K.P., Fleer A., Kimpen J.L., Van der Ent C.K. (2007). RSV mediates *Pseudomonas aeruginosa* binding to cystic fibrosis and normal epithelial cells. Pediatr. Res..

[11] Chattoraj S.S., Ganesan S., Jones A.M., Helm J.M., Comstock A.T., Bright-Thomas R., LiPuma J.J., Hershenson M.B., Sajjan U.S. (2011). Rhinovirus infection liberates planktonic bacteria from biofilm and increases chemokine responses in cystic fibrosis airway epithelial cells. Thorax.

[12] Kiedrowski M.R., Bomberger J.M. (2018). Viral-Bacterial Co-infections in the Cystic Fibrosis Respiratory Tract. Front. Immunol..

[13] Ortiz J.R., Neuzil K.M., Victor J.C., Wald A., Aitken M.L., Goss C.H. (2010). Influenza-associated cystic fibrosis pulmonary exacerbations. Chest.

[14] Somayaji R., Goss C.H., Khan U., Neradilek M., Neuzil K.M., Ortiz J.R. (2017). Cystic fibrosis pulmonary exacerbations attributable to respiratory syncytial virus and influenza: A population-based study. Clin. Infect. Dis..

[15] Kerem E., Viviani L., Zolin A., MacNeill S., Hatziagorou E., Ellemunter H., Drevinek P., Gulmans V., Krivec U., Olesen H. (2014). Factors associated with FEV1 decline in cystic fibrosis: Analysis of the ECFS patient registry. Eur. Respir. J..

[16] Dalbøge C.S., Hansen C.R., Pressler T., Høiby N., Johansen H.K. (2011). Chronic pulmonary infection with Stenotrophomonas maltophilia and lung function in patients with cystic fibrosis. J. Cyst. Fibros..

[17] 2023 Annual Data Report. http://www.ecfs.eu/ecfspr.

[18] Burgel P.-R., Southern K.W., Addy C., Battezzati A., Berry C., Bouchara J.-P., Brokaar E., Brown W., Azevedo P., Durieu I. (2024). Standards for the care of people with cystic fibrosis (CF); recognising and addressing CF health issues. J. Cyst. Fibros..

[19] Cleveland R.H., Stamoulis C., Sawicki G., Kelliher E., Zucker E.J., Wood C., Zurakowski D., Lee E. (2014). Brasfield and Wisconsin scoring systems have equal value as outcome assessment tools of cystic fibrosis lung disease. Pediatr. Radiol..

[20] Hafen G.M., Ranganathan S.C., Robertson C.F., Robinson P.J. (2006). Clinical scoring systems in cystic fibrosis. Pediatr. Pulmonol..

[21] van Ewijk B.E., van der Zalm M.M., Wolfs T.F.W., van der Ent C.K. (2005). Viral respiratory infections in cystic fibrosis. J. Cyst. Fibros..

[22] Hizal M., Yalcin E., Alp A., Ozden M., Karakaya J., Polat S.E., Tugcu G., Dogru D., Ozcelik U., Kiper N. (2020). Respiratory viruses: What is their role in acute exacerbations in children with cystic fibrosis?. Pediatr. Pulmonol..

[23] Wat D., Doull I. (2003). Respiratory virus infections in cystic fibrosis. Paediatr. Respir. Rev..

[24] Armstrong D., Grimwood K., Carlin J.B., Carzino R., Hull J., Olinsky A., Phelan P.D. (1998). Severe viral respiratory infections in infants with cystic fibrosis. Pediatr. Pulmonol..

[25] Hiatt P.W., Grace S.C., Kozinetz C.A., Raboudi S.H., Treece D.G., Taber L.H., Piedra P.A. (1999). Effects of viral lower respiratory tract infection on lung function in infants with cystic fibrosis. Pediatrics.

[26] Smyth R.L., Smyth A.R., Tong C.Y.W., Hart C.A., Heaf D.P. (1995). Effect of respiratory virus infections including rhinovirus on clinical status in cystic fibrosis. Arch. Dis. Child..

[27] Bilton D., Canny G., Conway S., Dumcius S., Hjelte L., Proesmans M., Tümmler B., Vavrova V., De Boeck K. (2011). Pulmonary exacerbation: Towards a definition for use in clinical trials. Report from the EuroCareCF Working Group on outcome parameters in clinical trials. J. Cyst. Fibros..

[28] Castellani C., Simmonds N.J., Barben J., Addy C., Bevan A., Burgel P.-R., Drevinek P., Gartner S., Gramegna A., Lammertyn E. (2023). Standards for the care of people with cystic fibrosis (CF): A timely and accurate diagnosis. J. Cyst. Fibros..

[29] Ramsey B.W., Gore E.J., Smith A.L., Cooney M.K., Redding G.J., Foy H. (1989). The Effect of Respiratory Viral Infections on Patients With Cystic Fibrosis. Arch. Pediatr. Adolesc. Med..

[30] Esther C.R., Lin F.C., Kerr A., Miller M.B., Gilligan P.H. (2014). Respiratory viruses are associated with common respiratory pathogens in cystic fibrosis. Pediatr. Pulmonol..

[31] Jones A.M. (2022). Infection control in cystic fibrosis: Evolving perspectives and challenges. Curr. Opin. Pulm. Med..

[32] VMeyer V.M.C., Siqueira M.M., Costa P.F.B.M., Caetano B.C., Lopes J.C.O., Folescu T.W., Motta F.D.C., Omri A. (2020). Clinical impact of respiratory virus in pulmonary exacerbations of children with Cystic Fibrosis. PLoS ONE.

[33] Gulla K.M., Balaji A., Mukherjee A., Jat K.R., Sankar J., Lodha R., Kabra S.K. (2019). Course of illness after viral infection in Indian children with cystic fibrosis. J. Trop. Pediatr..

[34] Scott J.E., O’Toole G.A. (2019). The yin and yang of Streptococcus lung infections in cystic fibrosis: A model for studying polymicrobial interactions. J. Bacteriol..

[35] Scherz V., Caruana G., Taffé P., Brouillet R., Bertelli C., Jaton K., Fougère Y., Posfay-Barbe K.M., Mornand A., Rochat-Guignard I. (2022). Unexpected associations between respiratory viruses and bacteria with Pulmonary Function Testing in children suffering from Cystic Fibrosis (MUCOVIB study). J. Cyst. Fibros..

[36] Hamed D.H., Soliman M.S., Emam O.S., El Attar M.M. (2022). Is there a role of viral infection in cystic fibrosis exacerbation in children?. Turk. J. Pediatr..

[37] Wat D., Gelder C., Hibbitts S., Cafferty F., Bowler I., Pierrepoint M., Evans R., Doull I. (2008). The role of respiratory viruses in cystic fibrosis. J. Cyst. Fibros..

[38] Gonzalez-Rosales N., Kasi A.S., McCracken C.E., Silva G.L., Starks M., Stecenko A., Guglani L. (2023). Impact of viral respiratory infections on pulmonary exacerbations in children with cystic fibrosis. Pediatr. Pulmonol..

[39] Carter S.C., Franciosi A.N., O’sHea K.M., O’cArroll O.M., Sharma A., Bell A., Keogan B., O’rEilly P., Coughlan S., Law S.M. (2022). Acute Pulmonary Exacerbation Phenotypes in Patients with Cystic Fibrosis. Ann. Am. Thorac. Soc..

[40] Hilliam Y., Armbruster C.R., Atteih S.E., Rapsinski G.J., Moore J., Koirala J., Krainz L., Gaston J., Williams J., Cooper V.S. (2025). Respiratory viral infection is associated with increased Pseudomonas abundance in cystic fibrosis airways. bioRxiv.

[41] Kartsiouni E., Chatzipanagiotou S., Tamvakeras P., Douros K. (2022). The role of viral infections in pulmonary exacerbations of patients with non-cystic fibrosis bronchiectasis: A systematic review. Respir. Investig..

[42] Sinha M., Zabini D., Guntur D., Nagaraj C., Enyedi P., Olschewski H., Kuebler W.M., Olschewski A. (2022). Chloride channels in the lung: Challenges and perspectives for viral infections, pulmonary arterial hypertension, and cystic fibrosis. Pharmacol. Ther..

[43] Londino J.D., Lazrak A., Jurkuvenaite A., Collawn J.F., Noah J.W., Matalon S. (2013). Influenza matrix protein 2 alters CFTR expression and function through its ion channel activity. Am. J. Physiol. Cell. Mol. Physiol..

[44] Londino J.D., Lazrak A., Noah J.W., Aggarwal S., Bali V., Woodworth B.A., Bebok Z., Matalon S. (2015). Influenza virus M2 targets cystic fibrosis transmembrane conductance regulator for lysosomal degradation during viral infection. ASEB J..

[45] Cao K., Chen M., Jie X., Wang Y., Li Q., Xu J. (2015). H5N1 virus hemagglutinin inhibition of cAMP-dependent CFTR via TLR4-mediated Janus tyrosine kinase 3 activation exacerbates lung inflammation. Mol. Med..

[46] Kim J.H., Kwon H.J., Jang Y.J. (2012). Effects of rhinovirus infection on the expression and function of cystic fibrosis transmembrane conductance regulator and epithelial sodium channel in human nasal mucosa. Ann. Allergy Asthma Immunol..

[47] Annual Reports|European Cystic Fibrosis Society (ECFS). https://www.ecfs.eu/projects/ecfs-patient-registry/annual-reports.

[48] Saluzzo F., Riberi L., Messore B., Loré N.I., Esposito I., Bignamini E., De Rose V. (2022). CFTR Modulator Therapies: Potential Impact on Airway Infections in Cystic Fibrosis. Cells.

[49] De Jong E., Garratt L.W., Looi K., Lee A.H., Ling K.-M., Smith M.L., Falsafi R., Sutanto E.N., Hillas J., Iosifidis T. (2021). Ivacaftor or lumacaftor/ivacaftor treatment does not alter the core CF airway epithelial gene response to rhinovirus. J. Cyst. Fibros..

[50] Thornton C.S., Caverly L.J., Kalikin L.M., Carmody L.A., McClellan S., LeBar W., Sanders D.B., West N.E., Goss C.H., Flume P.A. (2024). Prevalence and Clinical Impact of Respiratory Viral Infections from the STOP2 Study of Cystic Fibrosis Pulmonary Exacerbations. Ann. Am. Thorac. Soc..

[51] Fireizen Y., Ahmed M., Vigers T., Akong K., Ryu J., Hahn A., Fanous H., Koumbourlis A., Tirakitsoontorn P., Arrieta A. (2025). Changing Epidemiology of Pediatric Pulmonary Exacerbations in Cystic Fibrosis. Pediatr. Pulmonol..

