# Supplementary material for: Viral Infection Correlates with a Better Clinical Outcome than Pulmonary Exacerbation of Bacterial Origin in Paediatric Patients with Cystic Fibrosis
Source: Pathogens. 2025 Aug 26;14(9):850. doi: 10.3390/pathogens14090850 (PMC12472314; doi:10.3390/pathogens14090850)
Supplement: Supplementary file 1 [file pathogens-14-00850-s001.zip › pathogens-3796091-supplementary.pdf]

Supplementary Materials

Table S1. Pulmonary pathogen characteristics of patients included in the study.

Pathogen	PE		STABLE	
	**Viral-Negative (n = 30)**	**Viral-Positive (n = 19)**	**Viral-Negative (n = 10)**	**Viral-Positive (n = 6)**
Pseudomonas aeruginosa *	6 (20%)	4 (21.05%)	1 (10%)	1 (16.67%)
Staphylococcus aureus *	22 (73.33%)	16 (84.21%)	7 (70%)	6 (100%)
Achromobacter *	5 (16.67%)	1 (5.26%)	0	1 (16.67%)
Mycoplasma pneumoniae ^	0	0	0	0
Aspergillus spp. *	1 (3.33%)	2 (10.53%)	0	0

* Microorganisms present in sputum culture; ^ positive molecular tests.
